# Diagnostic Efficacy of Sentinel Lymph Node Biopsy in Early Oral Squamous Cell Carcinoma: A Meta-Analysis of 66 Studies

**DOI:** 10.1371/journal.pone.0170322

**Published:** 2017-01-20

**Authors:** Muyuan Liu, Steven J. Wang, Xihong Yang, Hanwei Peng

**Affiliations:** 1 Department of Head and Neck, Cancer Hospital of Shantou University Medical College, Shantou, China; 2 Department of Otolaryngology-Head and Neck Surgery, University of Arizona College of Medicine, Tucson, Arizona, United States of America; Texas A&M University, UNITED STATES

## Abstract

**Objectives:**

The diagnostic efficacy of sentinel lymph node biopsy(SLNB) in early oral squamous cell carcinoma(OSCC) still remains controversial. This meta-analysis was conducted to assess the diagnostic value of SLNB in clinically neck-negative T1-2 OSCC.

**Methods:**

A systematic literature search for relevant literature published up to September 11, 2016 was conducted in PubMed, Embase, Web of Science, Cochrane Library and ClinicalTrials, and the reference lists of eligible studies were examined. Data from different studies were pooled to estimate the summary sentinel lymph node(SLN) identification rate, sensitivity, negative predictive value. Summary receiver operator characteristic curve(SROC) was plotted and area under the SROC curve (AUC) was calculated to evaluate the overall diagnostic efficacy. Threshold effect was assessed with use of the spearman correlation coefficient. Between-study heterogeneity was tested using the Q tests and the *I*^2^ statistics. Subgroup analyses were conducted in view of the greater effect of different study characteristics on diagnostic efficacy of SLN. Deeks’ funnel plot asymmetry test was performed to evaluate publication bias. Sensitivity analysis was evaluated through omitting studies one by one and comparing the pooled results of random-effects model and fixed-effects model. All analyses were performed using Review Manager (version 5.3.5), Meta-DiSc (version 1.4), Comprehensive Meta Analysis (version 2.0) and STATA (version 12).

**Results:**

66 studies comprising 3566 patients with cT1-2N0 OSCC were included in this meta-analysis. The pooled SLN identification rate was 96.3%(95% CI: 95.3%-97.0%). The pooled sensitivity was 0.87 (95% CI: 0.85–0.89), pooled negative predictive value was 0.94 (95% CI: 0.93–0.95), and AUC was 0.98 (95% CI: 0.97–0.99). Subgroup analyses indicated that SLN assessment with immunohistochemistry(IHC) achieved a significantly higher sensitivity than without IHC.

**Conclusions:**

This meta-analysis suggests that SLNB has a high diagnostic accuracy in cT1-2N0 oral squamous cell carcinoma, and is an ideal alternative to elective neck dissection. Furthermore, the use of IHC can significantly improve SLNB diagnostic sensitivity for early OSCC.

## Introduction

Oral squamous cell carcinoma (OSCC) is one of the most common types of cancer in the world, with a considerable incidence of new cases every year. Approximately 50% of the patients with OSCC present with early stage disease(cT1-2N0) [1]. The main prognostic factor is occult lymph node metastasis in the neck. As it was reported in previous literatures, the overall rate of occult lymph node metastasis is 20%-30% in early stage OSCC patients [2–4]. Therefore, elective neck dissection remains the gold standard treatment in many institutions, resulting in overtreatment in over 70% of early OSCC patients and a considerable morbidity. For this reason, in recent years, sentinel lymph node biopsy(SLNB) has become more important and popular in the cervical treatment of patients with early OSCC. The sentinel lymph node (SLN) procedure is based on the theory that flow from a primary tumor travels sequentially to the sentinel lymph node and subsequently to the remaining lymph node basin [5]. Compared to elective neck dissection, SLNB is less invasive, cost-effective and beneficial to patient quality of life [6–9]. But the diagnostic efficacy of SLNB in early OSCC remains controversial [10–12]. Furthermore, most previous individual studies contained too small of a sample size to yield a valid conclusion. In addition, previous meta-analyses mainly focused on head and neck cancer or oral and oropharyngeal carcinoma [13–16]. However, combining different subset of head and neck cancer with differing clinical characteristics and metastasis patterns, can lead to heterogeneous results for SLNB. Although some previous meta-analyses have conducted subgroup analysis on OSCC, the small included sample size was underpowered to yield credible pooled findings. In recent years, many high quality prospective and some multi-institutional studies on the diagnostic efficacy of SLNB in early OSCC have been published [17–19]. Therefore, we performed a meta-analysis to summarize the diagnostic efficacy of SLNB specially focused exclusively on early OSCC. Additionally, we further stratified results by different clinical and study characteristics in order to explore the potential factors that may affect the diagnostic accuracy and applicability of SLNB.

## Materials and Methods

### Search strategy

We conducted a search for relevant literatures published up to September 11, 2016 in PubMed, Embase, Web of Science and Cochrane Library. The following medical subject headings(MeSH) and keywords were used: ("oral neoplasm" or "oral cancer" or "oral tumor" or "mouth neoplasm" or "mouth cancer" or "mouth tumor" or "head and neck neoplasm" or "head and neck cancer" or "head and neck tumor") and ("sentinel lymph node biopsy" or "sentinel"). We used no language restrictions. We also manually searched the reference lists of eligible studies and ClinicalTrials.gov to ensure identification of relevant published and unpublished studies.

### Inclusion and exclusion criteria

Articles included need to fulfill the following criteria: (1) Human cT1/T2N0 oral cavity squamous cell carcinoma patients (in studies that included T3, T4, N+ or other head and neck tumor cases, only the cT1–T2N0 oral cancer cases were selected); (2) the use of radioactive tracer, blue dye or indocyanine green; (3) presence of "gold standard", which was defined as the use of histological evaluation and follow-up; (4) studies presented sufficient data to allow for the construction of 2×2 tables, including true positive (TP), false positive (FP), false negative (FN) and true negative (TN); (5) Full text available in English. Studies that met the following criteria were excluded: (1) reports of duplicate data published in other studies; (2) letters, editorials, case reports or reviews; (3) studies without qualified data; (4) studies that included T3, T4 or N^+^ oral cavity cases or other head and neck tumors and not possible to be separated; (5) Full text in English unavailable.

Two reviewers(MY Liu and XH Yang) independently performed first-stage screening of titles and abstracts based on the research question. For the second screening, we retrieved articles in full text according to the initial screening. Any discrepancies were resolved by discussion or referred to a third author.

### Data extraction and quality assessment

Two investigators (MY Liu and XH Yang) independently reviewed the full texts of included studies and recorded the following data: first author, year of publication, sample size, description of study population (age), study design (prospective or retrospective), pathology (H&E staining, immunohistochemistry (IHC), serial sectioning (SS)), SLN tracer, SLN identification rate, average of SLNs harvested, data for diagnostic meta-analysis (TP, FP, FN, and TN) and so on. Results were then compared and any disagreements were settled by consensus. Concerning the quality of study design, study quality was assessed with the QUADAS-2 checklist for studies of diagnostic accuracy included in systematic reviews [20].

### Analysis

The identification rate, sensitivity and negative predictive value together with their 95% confidence intervals (95% CIs) were summarized in the current meta-analysis. The sensitivity and specificity of each included study were used to plot the summary receiver operator characteristic (SROC) curve and calculate the area under the SROC curve (AUC).

Q tests and *I*^2^ statistics were used to assess the degree of heterogeneity between studies. A *p* value less than 0.1 for the Q test and an *I*^2^ higher than 50% indicated the existence of significant heterogeneity. Pooled estimates were derived using the fixed-effects model if significant heterogeneity was not present. In case of heterogeneity, the random-effects model was applied.

We assessed diagnostic threshold effect with use of the spearman correlation coefficient. In addition, We further stratified results by the average of SLNs harvested (low: <2, medium: 2≤ and <3 or high: ≥3), SLN pathology methods(IHC or not, SS or not), type of reference test(neck dissection or follow-up), SLN tracer(single tracer or multiple tracers), study design (prospective or retrospective) and publication year(early: 2000–2008 or late: 2009–2016) in view of the greater effect of different study characteristics on diagnostic efficacy of SLN, and to explore the sources of between-study heterogeneity.

In this meta analysis, we performed a sensitivity analysis to evaluate the credibility and consistency of the results through omitting studies one by one and comparing the pooled results of random-effects model and fixed-effects model. Publication bias was assessed by using Deeks' funnel plot.

Two-sided *p* values were calculated with *p*<0.05 considered significant for all tests. We did statistical analysis with Review Manager (version 5.3.5), Meta-DiSc (version 1.4), Comprehensive Meta Analysis (version 2.0) and STATA (version 12).

## Results

### Search results and study selection

**Fig 1:**
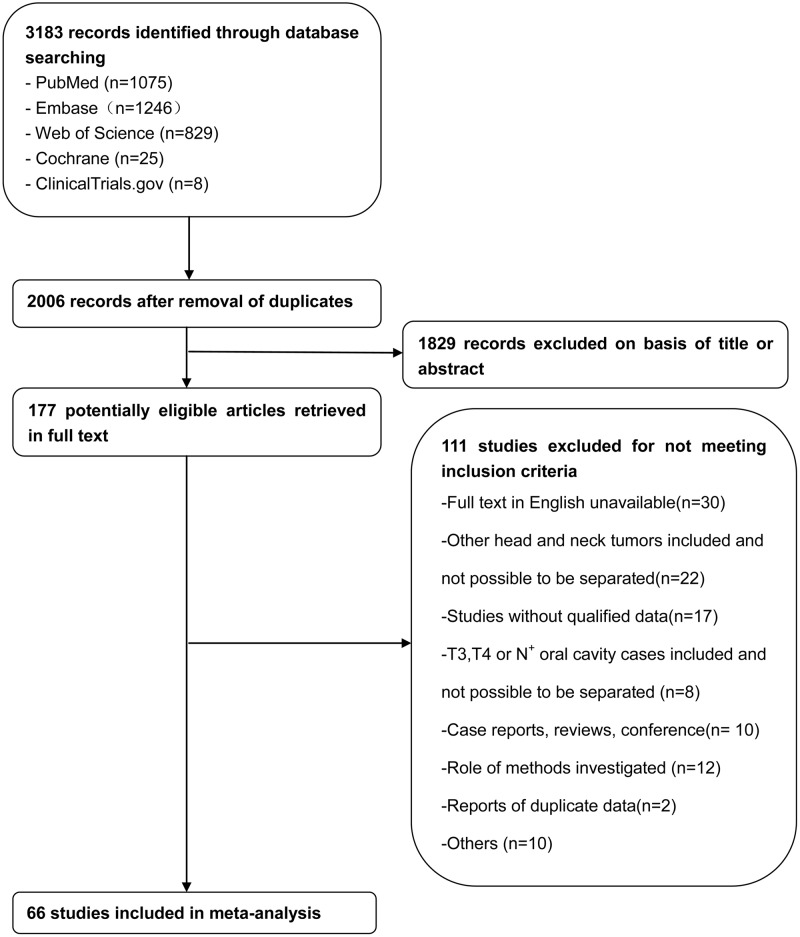
Study flow diagram.

Fig 1 shows the study flowchart. The initial search returned a total of 3183 studies, of which 1177 were excluded as duplications. The remaining 2006 articles were subject to further evaluation. After titles and abstracts were reviewed, 1829 were excluded, leaving 177 articles available for full text review. After full text review, an additional 111 manuscripts were excluded (the reasons were presented on Fig 1). Finally, 66 studies fulfilled the inclusion criteria for the meta analysis, comprising 3566 patients [5, 10–12, 17–19, 21–79] (Fig 1).

### Study characteristics

Table 1 summarizes individual studies and their characteristics. Of the 66 studies, the publication years of the included articles ranged from 2000 to2016 (26 articles were published during 2000 and 2008 while 40 articles were published during 2009 and 2016). Among the 66 studies, 56 researches were prospective while 10 studies were retrospective. Additionally, 43 studies detected the SLN by single tracer while 23 studies by multiple tracers. In all included studies, SLN was diagnosed based on histopathology(H&E staining, IHC and/or SS), which is considered the gold standard reference for SLN metastasis diagnosis. Of the total 3566 cases, SLN could be harvested in 3516 cases. The pooled SLN identification rate was 96.3%(95% CI: 95.3%-97.0%). The data of average SLN harvested per person was reported in 38 studies. The TP, FP, FN and TN results for individual studies were shown in Table 1.

**Table 1:** Basic characteristics of included studies.

study	year	Design	Population		Index Test			Reference Test ND or FU(mean, range)	Outcome					
			N	Age, median (range) or mean(SD), y	SLN tracer	SLN localization	Pathology (SLN)		SLN identification rate	No. of SLN, mean	TP	FP	FN	TN
Ramamurthy	2014	Pros	32	43(26–70)	B	B	H+I+S	ND	29/32	1.56	4	0	1	24
Chung	2015	Pros	61	49.3(10.3)	R	L+G	H+I+S	FU (70months, 49–111)	61/61	NR	12	0	5	44
Julio	2007	Pros	14	65.9(13.7)	R+ICG	L+G	H+I	ND	14/14	3.2	3	0	0	11
Heuveling	2014	Pros	66	NR	R+B	L+G+B+SPECT	Unclear	ND	66/66	2	11	0	2	53
Terada	2011	Pros	45	62(30–85)	R	L+G+SPECT	H	FU (46months, 9–72)	45/45	NR	5	0	5	35
Barzan	2002	Pros	10	64(36–85)	R	G	Unclear	ND	9/10	NR	2	0	0	7
Bluemel	2014	Pros	23	58.7(13)	R	L+G+SPECT/CT	H+I+S	ND	23/23	3.1	5	0	0	18
Terada	2008	Pros	43	NR	R	L+G+SPECT/CT	Unclear	ND	41/43	NR	5	0	1	35
Chiesa	2000	Pros	11	NR	R	L+G	Unclear	ND	10/11	NR	3	0	0	7
Dequanter	2013	Pros	20	64(?)	R	L+G	H+I+S	FU (59months, ?-?)	20/20	NR	4	0	0	16
Broglie	2011	Pros	69	60(?)	R	L+G+SPECT+PET-CT	Unclear	FU (59.8months, 2.6–120.7)	69/69	NR	23	0	2	44
Vigili	2007	Pros	12	57.4(?)	R	L+G	H+I	ND	12/12	2.1	5	0	0	7
Aida	2014	Pros	25	61.2(42–82)	R	L+G+SPECT/CT	Unclear	FU (?months, 7–88)	25/25	NR	8	0	0	17
Joost	2013	Pros	7	59.5(33–73)	ICG	NI	H	ND	7/7	1.7	2	0	1	4
Bilde	2008	Pros	51	58(29–90)	R	L+G+SPECT/CT	H+I+S	ND	51/51	3	11	0	0	40
Chaturvedi	2015	Pros	53	44(29–70)	R	L+G+SPECT	H	ND	51/53	3.8	10	0	4	37
Yen	2006	Pros	25	47.8(30–66)	R	L+G	H+I+S	ND	24/25	2.4	6	0	0	18
Minamikawa	2005	Retro	18	NR	B	B	Unclear	FU (NR)	15/18	NR	4	0	1	10
Ram	2015	Retro	42	61.3(40–83)	R	L+G+SPECT/CT	H+I+S	FU (NR)	42/42	NR	8	0	2	32
Schilling	2015	Pros	415	61(28–92)	R+B	L+G+B	H+I+S	FU (>36months)	415/415	3.2	94	0	15	306
Civantos	2010	Pros	140	58(24–90)	R	L+G	H+I	ND	140/140	3	37	0	4	99
Rigual	2013	Retro	38	62(14)	R	L+G	H	FU (31months, 3–71)	38/38	2	5	0	2	31
Tartaglione	2016	Pros	434	NR	R+B	L+G+B+SPECT/CT	H+I+S	ND	434/434	3.2	105	0	14	315
Harri	2008	Pros	13	65(30–84)	R+B	L+G+B	H+I+S	FU (21months, 12–42)	13/13	3.1	2	0	0	11
Pezier	2012	Pros	59	62.5(38–90)	R+B	L+G+B	H+I	FU (22.5months, 0.26–53)	57/59	2.6	17	0	1	39
Hart	2005	Pros	12	62.75(35–83)	R	L+G	H+I+S	ND	12/12	NR	2	0	0	10
Keyvan	2016	Pros	10	52(21–82)	R	L+G	Unclear	ND	10/10	2.4	3	0	0	7
Flach	2014	Pros	62	61.2(28.8–82.6)	R+B	L+G+B	H+I+S	FU (52.5months, 5.3–76.7)	62/62	NR	20	0	5	37
Vishno	2015	Pros	65	47(20–77)	B	B	H+I	ND	60/65	2.02	7	0	1	52
Hasegawa	2011	Pros	61	NR	R	G	Unclear	FU (NR)	60/61	NR	10	0	3	47
Burns	2009	Pros	9	59.2(38–80)	R+B	L+G+B	H+I	FU (?months, 9–24)	9/9	1.3	3	0	0	6
Kontio	2004	Pros	15	63.8(35–81)	R+B	L+G+B	H+I	ND	15/15	2.8	3	0	0	12
Frerich	2007	Pros	26	NR	R	G	H+I+S	FU (27.5months, 7.2–49.5)	26/26	2.1	8	0	2	16
Hiraki	2016	Retro	47	65.4(12.6)	R	L+G+SPECT/CT	H+S	FU (38.5months, 12.4–64.6)	47/47	2.1	9	0	2	36
Honda	2015	Pros	31	64(33–91)	B	B+CT(Iopamidol)	Unclear	FU (>30months)	28/31	NR	4	0	1	23
Fan	2014	Retro	30	48(27–75)	R+B	L+G+B	H	FU (>120months)	30/30	NR	9	0	1	20
Rigual	2005	Pros	20	NR	R+B	L+G+B	H	ND	20/20	NR	10	0	2	8
Stoeckli	2001	Pros	15	56(36–81)	R+B	L+G+B	H+I+S	ND	15/15	NR	3	0	0	12
Terada	2006	Pros	20	NR	R	L+G+SPECT/CT	H	FU(NR)	20/20	3.3	6	0	0	14
Jeong	2006	Pros	20	53(35–68)	R	L+G	H+I+S	ND	20/20	2.55	6	0	0	14
Thomsen	2007	Pros	39	?(32–90)	R+B	L+G+B	H+I+S	FU (28months, 4–54)	37/39	NR	11	0	0	26
Hoft	2004	Pros	20	NR	R	L+G	H+I+S	ND	20/20	3.2	6	0	0	14
Samant	2014	Pros	34	61(24–82)	R+B	L+G+B	H+I	FU(36months, 2–60)	32/34	NR	6	0	2	24
Toom	2015	Retro	90	60(29–86)	R+B	L+G+B+SPECT/CT	H+I+S	FU(18months, 2–62)	87/90	2	26	0	2	59
Yoshimoto	2012	Retro	145	63(21–92)	R	L+G+SPECT/CT	H+I	FU (NR)	145/145	2.9	24	0	7	114
Stoeckli	2007	Pros	79	58.5(34–87)	R	L+G	H+I+S	FU (19months, 3–40)	78/79	NR	29	0	2	47
Melkane	2012	Pros	174	56(28–86)	R	L+G	H+I+S	FU (>36months)	166/174	2	42	0	6	118
Civantos	2003	Pros	14	62(34–79)	R	L+G+PET maping	H+I	ND	14/14	NR	6	0	1	7
Alvarez	2014	Pros	28	61.2(41–87)	R+B	L+G+B	H+I+S	FU (>60months)	28/28	NR	7	0	4	17
Peng	2015	Pros	19	60.5(43–77)	B+ICG	B+NI	H	ND	19/19	3.4	3	0	0	16
Hernando	2016	Pros	32	66.4(40–90)	R	L+G	H+I+S	FU (48.2months, 7–70)	32/32	2	3	0	3	26
Ikram	2013	Pros	42	52(31–75)	R	L+G	H+I+S	ND	38/42	NR	7	0	0	31
Taylor	2001	Pros	8	61.9(22–80)	R	L+G	Unclear	ND	8/8	NR	3	0	0	5
Yamauchi	2012	Pros	11	62.3(36–84)	R	L+G	H+S	FU (37.1months, 20.1–54.1)	11/11	NR	2	0	1	8
Thomsen	2005	Pros	40	?(32–90)	R+B	L+G+B	H+I+S	FU (15.8months, 4.3–40.4)	40/40	NR	11	0	3	26
Pedersen	2016	Retro	253	63(30–95)	R	L+G+SPECT/CT	H+I+S	FU (32months, 1–92)	253/253	3	68	0	9	176
Kaya	2015	Pros	18	54.5(28–76)	R	L+G	H+I	ND	18/18	NR	5	0	0	13
Tartaglione	2008	Pros	22	62.6(28–80)	R	L+G	H+I	FU (23.2months, 10–35)	22/22	2.2	8	0	0	14
Hernandez	2005	Pros	48	57(28–83)	R+B	L+G+B	H+S	ND	48/48	2	10	0	3	35
Bilde	2006	Pros	34	58(47–70)	R	L+G+SPECT/CT	H+I+S	ND	32/34	3	6	0	0	26
Bell	2013	Pros	36	60.5(20–87)	R	L+G	H+I+S	ND	35/36	1.9	7	0	1	27
Matsuzuka	2014	Retro	29	66(31–82)	R	L+G	H	FU (91months, 10–165)	29/29	3.1	6	0	2	21
Heuveling	2012	Retro	60	60(29–81)	R+B	L+G+B	H+I+S	FU (19months, 5–51)	60/60	3	21	0	1	38
Agrawal	2015	Pros	66	60.8(12.8)	R	L+G+SPECT/CT	H+I	ND	65/66	3.9	30	0	1	34
Nakamura	2015	Pros	15	63.1(44–84)	R+ICG	L+G+NI	Unclear	FU (38.5months, 10.7–69.9)	15/15	2.2	1	0	1	13
Mozzillo	2001	Pros	41	NR	R+B	L+G+B	Unclear	ND	39/41	NR	4	0	0	35

Abbreviations: Pros, Prospective; Retro, Retrospective; NR, Not reported; R, Radionucleotide; B, Blue dye; ICG, Indocyanine green; NI, near-infrared fluorescence camera; H, Hematoxylin and eosin; I, Immunohistochemistry; S, Serial sectioning; ND, Neck dissection; FU, Follow-up; SLN, Sentinel lymph node; TP, Ture positive; FP, False positive; FN, False Negative; TN, Ture negative.

### Quality of included studies

**Fig 2:**
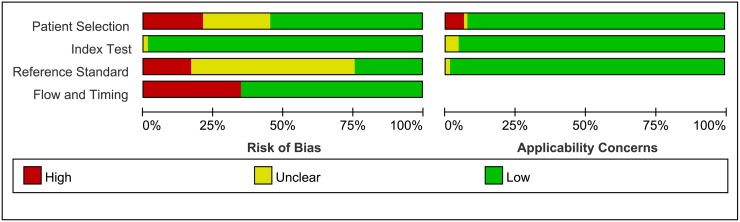
Results of QUADAS-2, Risk of bias and concerns regarding applicability.

Quality assessments are shown in a bar graph of QUADAS-2 in Fig 2. The graph indicates that all included studies were of moderately high quality. Risk of bias regarding patient selection was high in 14 (21.2%) studies mostly due to their retrospective nature without a consecutive or random sample enrollment of patients. Risk of bias regarding index test was unclear in only 1(1.5%) study while 65 (98.5%) studies were low risk. By contrast, the reference standard was unclear in 39 (59.1%) studies because in most of these studies it was unclear whether the index test and reference test were interpreted independently and blindly from each other. For risk of bias in flow and timing there were 23 (34.8%) studies considered high risk mainly due having a the different reference standard. In these studies, patients with positive SLN would undergo a comprehensive neck dissection and pathology results of cervical lymph nodes were the "gold standard" however SLN-negative patients would not undergo neck dissection and clinical follow-up was the "gold standard". There was less concern about the applicability of the studies. In 5 (7.6%) studies, there were concerns about applicability because of patient selection, in 3 (4.5%) studies because of the index test and in1 (1.5%) study because of the reference test.

### Diagnostic accuracy

**Fig 3:**
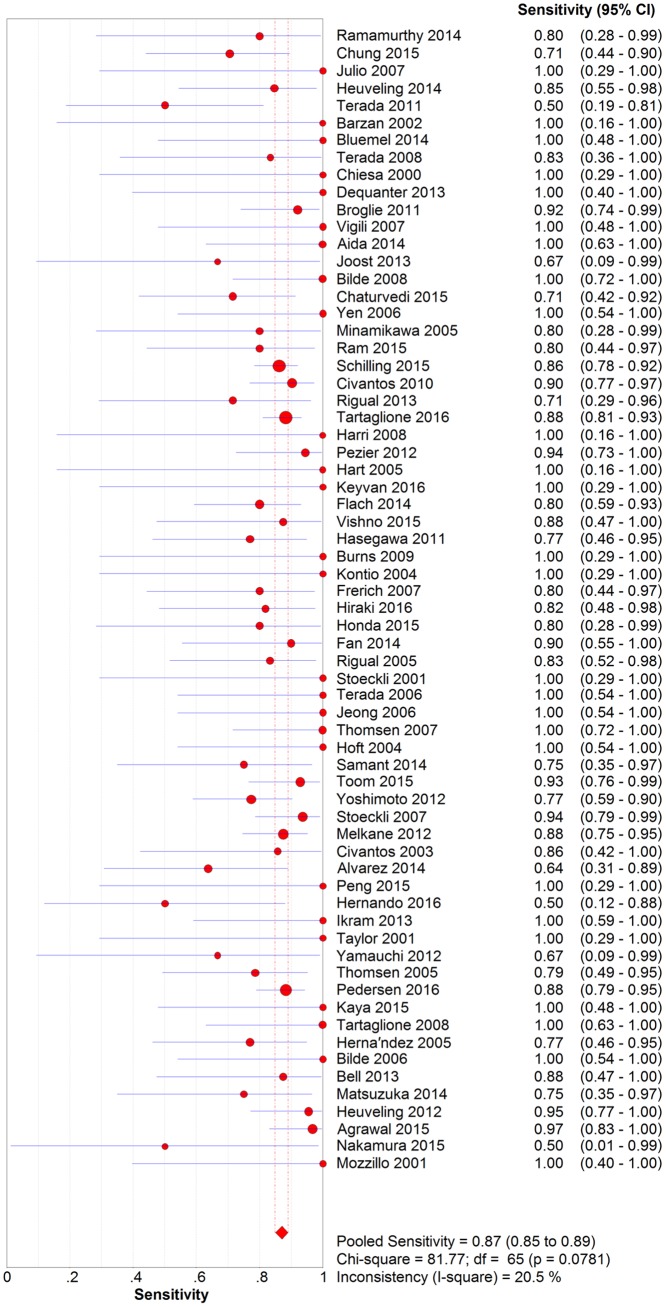
Forest plot of pooled sensitivity.

**Fig 4:**
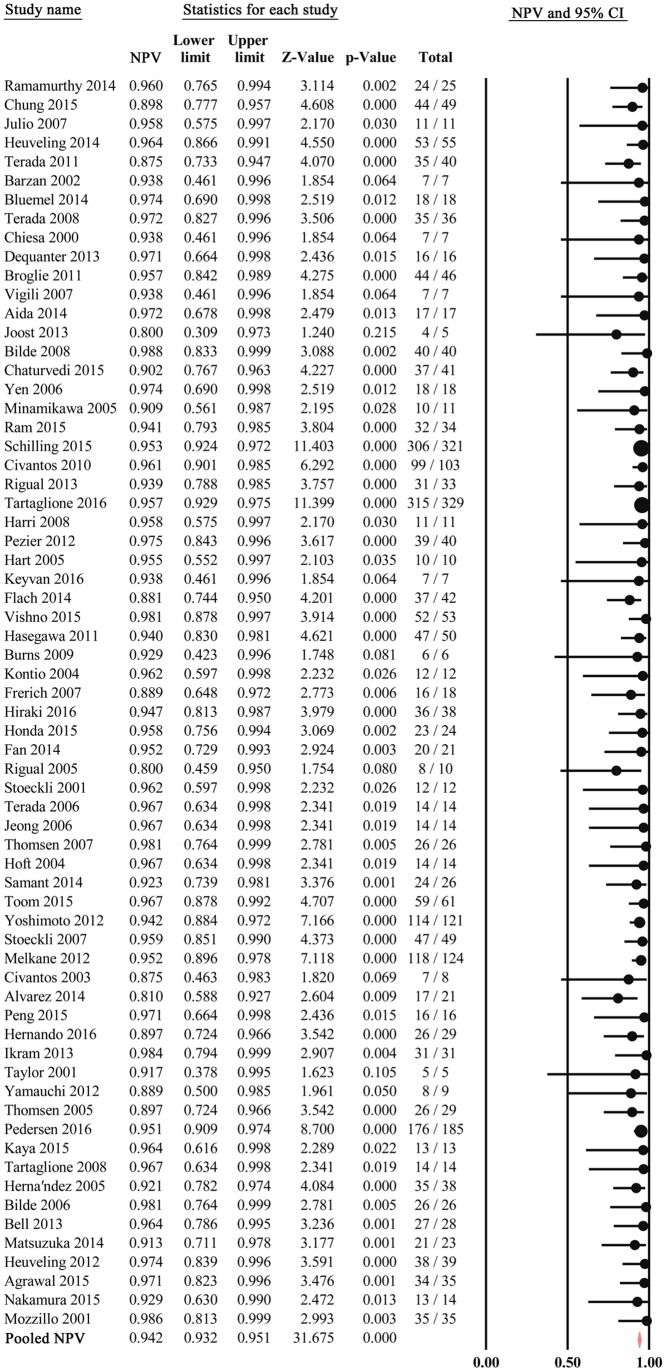
Forest plot of pooled negative predictive value.

**Fig 5:**
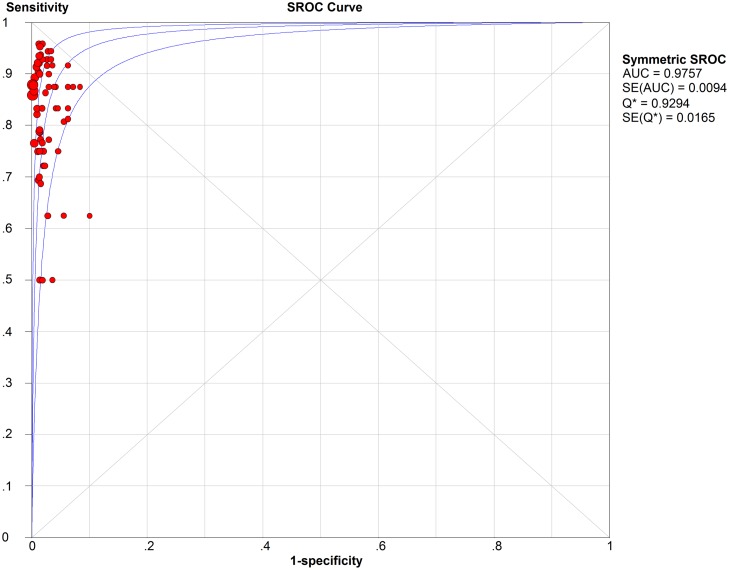
SROC curve.

Analysis of diagnostic threshold showed that the spearman correlation coefficient was -0.037 with a *p*-value of 0.769. Forest plots of data from the 66 studies on the sensitivity and negative predictive value of SLNB are shown in Figs 3 and 4, respectively. Since no significant heterogeneity were found between studies in sensitivity and negative predictive value data (*I*^2^ = 20.5% and *I*^2^ = 0.0, respectively), the fixed effects model was used to calculate the pool estimates in this study. In the present analysis, the pooled SLN identification rate, pooled sensitivity and negative predictive value were 96.3%(95% CI: 95.3%-97.0%), 0.87 (95% CI: 0.85–0.89) and 0.94 (95% CI: 0.93–0.95), respectively. Fig 5 shows the corresponding overall SROC curve with an AUC of 0.98 (95% CI: 0.97–0.99). In order to view the greater effect of different study characteristics on the diagnostic efficacy of SLN, subgroup analysis was conducted.

### Subgroup analysis

We conducted subgroup analysis by the average of SLNs harvested (low: <2, medium: 2≤ and <3 or high: ≥3), SLN pathology methods(IHC or not, SS or not), type of reference test (neck dissection or follow-up), SLN tracer (single tracer or multiple tracers), study design (prospective or retrospective) and publication year (early: 2000–2008 or late: 2009–2016). The pooled sensitivity, negative predictive value and AUC for each subgroup are listed in Table 2.

**Table 2:** Summary of subgroup analysis by different clinical characteristics.

Subgroup	Study (n)	Sensitivity [95% CIs]	NPV [95% CIs]	AUC [95% CIs]
IHC				
No	12	0.77 [0.68–0.85]	0.91[0.87–0.94]	0.97[0.95–0.99]
Yes	41	0.88 [0.86–0.90]	0.95[0.94–0.96]	0.98[0.97–0.99]
SS				
No	21	0.88[0.84–0.91]	0.93[0.91–0.95]	0.96[0.93–0.99]
Yes	32	0.87[0.84–0.90]	0.94[0.93–0.95]	0.98[0.97–0.99]
Average SLNs				
Low (N<2)	4	0.84 [0.60–0.97]	0.94[0.83–0.98]	0.90[0.75–1.00]
Medium(2≤N<3)	18	0.86 [0.81–0.90]	0.95[0.93–0.96]	0.98[0.97–1.00]
High(N≥3)	16	0.88 [0.86–0.92]	0.95[0.94–0.97]	0.98[0.97–0.99]
Publication Year				
Early(2000–2008)	26	0.92 [0.87–0.95]	0.94[0.91–0.96]	0.98[0.97–0.99]
Late(2009–2016)	40	0.86 [0.83–0.88]	0.94[0.93–0.95]	0.98[0.96–0.99]
SLN Tracer				
Single	43	0.87 [0.84–0.90]	0.94[0.93–0.95]	0.98[0.97–0.99]
Multiple	23	0.87 [0.84–0.90]	0.94[0.93–0.96]	0.96[0.93–0.99]
Study Design				
Prospective	56	0.87 [0.85–0.90]	0.94[0.93–0.95]	0.98[0.97–0.99]
Retrospective	10	0.86 [0.81–0.91]	0.95[0.93–0.96]	0.97[0.92–1.00]
Reference Test				
ND	32	0.90 [0.87–0.93]	0.95[0.94–0.96]	0.97[0.95–0.98]
FU	34	0.85 [0.82–0.88]	0.94[0.92–0.95]	0.98[0.97–0.99]

ND: neck dissection; FU: follow-up; IHC: immunohistochemistry; SS: Serial sectioning; NPV: negative predictive value.

For subgroup analyses conducted by the average of SLNs harvested, SLN tracer, study design and serial sectioning, no significant difference could be found among these groups. However, subgroup analysis based on immunohistochemistry(IHC) indicated that H&E combined with IHC was significantly more sensitive than single H&E staining with a sensitivity of 0.88(95%CI: 0.86–0.90) versus 0.77(95%CI: 0.68–0.85). Moreover, early publication subgroup and neck dissection subgroup yielded a better pooled sensitivity than late publication subgroup and clinical follow-up subgroup(0.92[0.87–0.95] *vs*. 0.86[0.83–0.88] and 0.90[0.87–0.93] *vs*. 0.85[0.82–0.88], respectively).

### Sensitivity analysis and publication bias

We performed sensitivity analyses to assess the credibility and consistency of the results through: (1)Omitting studies one by one. In the current meta analysis, with removal of any single study the pooled findings were essentially unchanged. (2) When switched the fixed-effects model to random-effects model, the pooled findings didn't change significantly. The sensitivity analyses supported the result was robustness.

**Fig 6:**
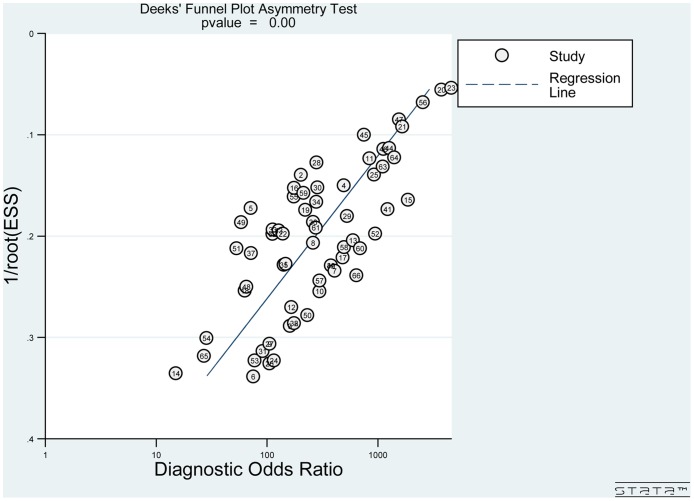
Deeks' funnel plot with regression line.

In order to evaluate potential publication bias, the Deeks' funnel plot asymmetry test was used. The slope coefficient was associated with a *P* value of 0.00 (Fig 6), revealed a likelihood of publication bias.

## Discussion

To our knowledge, this is the largest meta-analysis focused on the diagnostic efficacy of sentinel lymph node biopsy in early oral squamous cell carcinoma. In this meta-analysis of 66 studies comprising more than 3500 patients, SLNB yielded a pooled identification rate of 96.3%(95% CI: 95.3%-97.0%), a pooled sensitivity of 0.87(95%CI: 0.85–0.89), a pooled negative predictive value of 0.94 (95% CI: 0.93–0.95) and an AUC of 0.98 (95% CI: 0.97–0.99). The high pooled negative predictive value implied that only 6% of SLN-negative early oral cavity cancer patients would result in a false-negative regional recurrence during follow-up. This is similar to the regional recurrence rate after elective neck dissection in clinically neck-negative early OSCC reported by previous literature [80], and is far lower than the acceptable threshold of 20% cervical lymph node metastasis rate for prophylactic neck dissection. Therefore, elective neck dissection could be omitted in SLN-negative early OSCC patients. Moreover, the pooled sensitivity implies that 87% of occult cervical lymph node metastases could be diagnosed by SLNB and the false-negative rate is 13%. The occult lymph node metastasis rate has been reported to be 20%-30% for cT1-2N0 OSCC [2–4]. Therefore, we can estimate that SLNB applied to all early OSCC patients would result in a 2.6%-3.9% regional recurrence rate. This regional recurrence rate is acceptable when considering the serious complications and 70% overtreatment rate in traditional prophylactic neck dissection procedure. Overall, these pooled findings indicated that SLNB had an ideal diagnostic accuracy for predicting occult cervical lymph node metastases in early oral cancer patients and was an ideal alternative to neck dissection. In the previous meta-analyses focusing on the diagnostic efficacy of SLNB in head and neck cancer or oral/oropharyngeal cancer, Tim reported a pooled sensitivity of 0.92 (95%CI: 0.86–0.95) in oral cancer subgroup(n = 508), while Thompson reported a pooled sensitivity and negative predictive value of 0.94 (95%CI: 0.89–0.98) and 0.96 (95%CI: 0.93–0.99) respectively in the subset of oral cavity tumors(n = 631) [14, 15]. Compared to these previous meta-analyses, our research found a lower sensitivity of 0.87(95%CI: 0.85–0.89)(n = 3506). Since those two meta-analyses were published many years ago, we further stratified our results by publication year and found that the pooled sensitivity of early publications(2000–2008) in current meta-analysis was 0.92(95%CI: 0.87–0.95), more similar to the results reported by previous meta-analyses, and better than late publications(2009–2016). A possible reason for this difference may be that SLNB researches in early publications were still during the validation stage, and elective neck dissection of levels I-III was the gold standard for SLN-negative cases in most of these publications(69.2%, 18/26). But in more recent publications, most SLNB research studies use clinical follow-up as their gold standard for SLN-negative cases and only 35%(14/40) of studies were still using elective neck dissection(levels I-III) as their gold standard. Thus, we speculate that: (1) there may have occult lymph node metastases in level IV, level V or even contralateral neck that would be missed by the elective neck dissections in most of the earlier publications, resulting in an overestimated sensitivity; (2) SLNB with neck dissection is definitely easier than SLNB without neck dissection and this may also lead to a higher pooled sensitivity in the validation stage.

Based on our subgroup analyses, we found that SLNB with IHC yielded significantly better sensitivity than the no IHC subgroup. The pooled sensitivity was 0.88(95%CI: 0.86–0.90) in IHC subgroup but only 0.77(95%CI: 0.68–0.85) in the no IHC subgroup. These results indicated that application of IHC associated with a 11% relative increase in sensitivity. Based on this result, we strongly recommend that IHC should be performed for SLN pathologic analysis. By contrast, no significant difference could be found between serial sectioning subgroup and no serial sectioning subgroup. The pooled sensitivity was 0.88(95%CI: 0.84–0.91) and 0.87(95%CI: 0.84–0.90), respectively. A prospective study conducted by Bell demonstrated that SLNB performed with the use of routine H&E staining and IHC could accurately predict neck stage in early oral squamous cell carcinoma with a negative predictive value of 96% and that serial sectioning might not be necessary [75]. Meanwhile, routine serial sectioning was also deemed not feasible or practical to make a quick diagnosis for SLN during frozen section. In the current meta-analysis, our results confirmed Bell's conclusion.

Several limitations should be considered while interpreting our results. First, although we tried to incorporate all relevant studies, the Deeks' funnel plot still revealed a likelihood of publication bias. It is possible that we may have missed some eligible studies in our screening process. There may also have been small trials with opposite results that were never published. Second, quality assessment showed that there was high risk of bias in flow and timing because not all patients received the same reference standard. This bias might restrict interpretation of the true diagnostic efficacy of SLNB. Third, in almost all of the included studies, the SLNs were assessed by postoperative pathological procedure but not by frozen section. This might result in overestimating the practical clinical applicability of SLNB. Nevertheless, this didn't affect the validity of our pooled findings. Finally, similar to other meta-analyses, we included studies with different characteristics and designs. Nevertheless, the heterogeneity test and sensitivity analyses proved our pooled findings to be credible and consistent. Notwithstanding the limitations listed above, our meta-analysis also has its strengths: (1) this is the largest meta-analysis of the diagnostic efficacy of SLNB specifically focused on early oral squamous cell carcinoma; (2) by dividing studies into two subgroups based on the use of IHC, we confirmed that SLN assessment with IHC achieved a significantly higher sensitivity than without IHC; (3) Moreover, serial sectioning does not seem necessary for SLN assessment.

## Conclusions

Our results confirmed that SLNB had a high diagnostic accuracy in cT1-2N0 oral squamous cell carcinoma, and was an ideal alternative to elective neck dissection. We also found that H&E with IHC yielded much better diagnostic sensitivity than H&E alone. However, further clinical trials are required to verify the clinical utility and application of SLNB by frozen section but not by postoperative pathological assessment. In particular, further studies on the diagnostic accuracy of automated quantitative real-time PCR (qRT-PCR) assay for intra-operative SLN frozen section are required [81, 82].

## Supporting Information

S1 Appendix Search Strategy. (DOCX)

S2 Appendix Methodological Quality Summary. (TIF)

S3 Appendix PRISMA 2009 Checklist. (DOC)
