# Supplementary material for: Diagnostic Efficacy of Sentinel Lymph Node Biopsy in Early Oral Squamous Cell Carcinoma: A Meta-Analysis of 66 Studies
Source: PLoS One. 2017 Jan 20;12(1):e0170322. doi: 10.1371/journal.pone.0170322 (PMC5249063; doi:10.1371/journal.pone.0170322)
Supplement: S1 Appendix — (DOCX) [file pone.0170322.s001.docx]

**S1** **Appendix. Search Strategy**

**PUBMED**

01. "tongue neoplasms"[MeSH Terms]

02. "tongue neoplasms"

03. "tongue neoplasm"

04. "neoplasm, tongue"

05. "cancer of tongue"

06. "tongue cancers"

07. "tongue cancer"

08. "cancer, tongue"

09. "cancers, tongue"

10. "cancer of the tongue"

11. "tongue tumor"

12. "oral neoplasms"[MeSH Terms]

13. "oral neoplasms"

14. "oral neoplasm"

15. "neoplasms,oral"

16. "neoplasm,oral"

17. "oral cancer"

18. "cancer,oral"

19."cancers,oral"

20. "oral cancers"

21. "oral tumor"

22. "cancers of the mouth"

23. "mouth neoplasm"

24. "neoplasms,mouth"

25. "neoplasm,mouth"

26. "cancer of mouth"

27. "mouth cancers"

28. "mouth cancer"

29. "cancer,mouth"

30. "cancers,mouth"

31. "mouth tumor"

32. "head and neck neoplasms"[MeSH Terms]

33. "head and neck neoplasms"

34. "cancer of head and neck"

35. "head and neck cancer"

36. "cancer of the head and neck"

37. "head and neck tumor"

38. 1 or 2 or 3 or 4 or 5 or 6 or 7 or 8 or 9 or 10 or 11 or 12 or 13 or 14 or 15 or 16 or 17 or 18 or 19 or 20 or 21 or 22 or 23 or 24 or 25 or 26 or 27 or 28 or 29 or 30 or 31 or 32 or 33 or 34 or 35 or 36 or 37

39. "sentinel lymph node biopsy"[MeSH Terms]

40. "sentinel lymph node biopsy"

41. "biopsy, sentinel lymph node"

42. "lymph node biopsy, sentinel"

43. "sentinel"

44.39 or 40 or 41 or 42 or 43

45.38 and 44
