# Supplementary figures and images for: Diagnostic Efficacy of Sentinel Lymph Node Biopsy in Early Oral Squamous Cell Carcinoma: A Meta-Analysis of 66 Studies
Source: PLoS One. 2017 Jan 20;12(1):e0170322. doi: 10.1371/journal.pone.0170322 (PMC5249063; doi:10.1371/journal.pone.0170322)

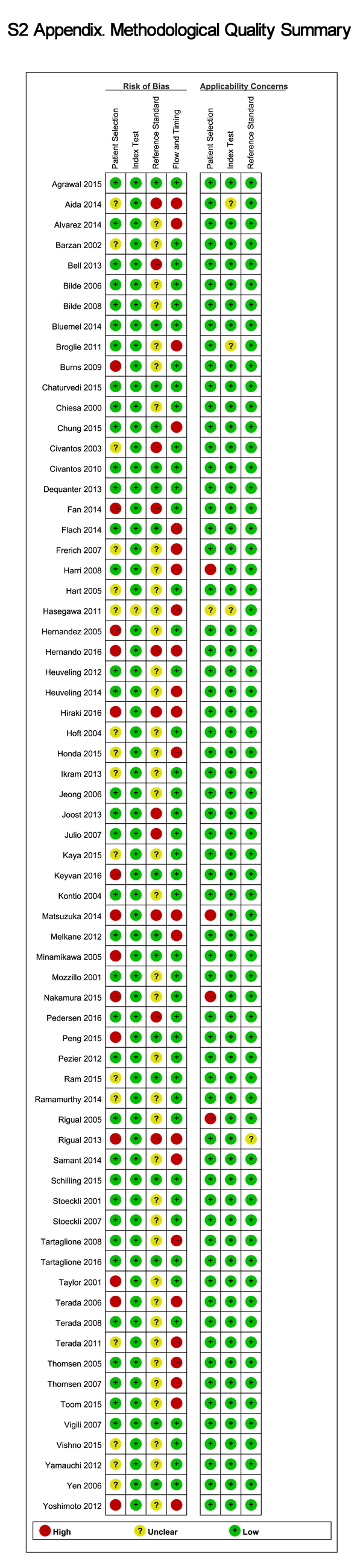

Supplement: S2 Appendix — (TIF) [file pone.0170322.s002.tif]
