# Supplementary material for: Diagnostic Efficacy of Sentinel Lymph Node Biopsy in Early Oral Squamous Cell Carcinoma: A Meta-Analysis of 66 Studies
Source: PLoS One. 2017 Jan 20;12(1):e0170322. doi: 10.1371/journal.pone.0170322 (PMC5249063; doi:10.1371/journal.pone.0170322)
Supplement: S3 Appendix — (DOC) [file pone.0170322.s003.doc]

| **Section/topic** | **#** | **Checklist item** | **Reported on page #** |
| --- | --- | --- | --- |
| **TITLE** | | |  |
| Title | 1 | The report is identified as a meta-analysis. | 1 |
| **ABSTRACT** | | |  |
| Structured summary | 2 | The summary includes background, objective, data sources, participants, study synthesis methods, results and conclusions. | 2-3 |
| **INTRODUCTION** | | |  |
| Rationale | 3 | Described in the introduction. | 4-5 |
| Objectives | 4 | Stated in the introduction. | 4-5 |
| **METHODS** | | |  |
| Protocol and registration | 5 | The protocol is described in the Methods. Registration does not apply. | 6 |
| Eligibility criteria | 6 | Study characteristics and report characteristics were used as criteria for eligibility rationale. | 6 |
| Information sources | 7 | All information sources and date last searched were described in the search. | 6 |
| Search | 8 | Full electronic search strategy for at least one database was presented and it could be repeated. | 6 and Supporting Informationl file 3 |
| Study selection | 9 | The process for selecting studies was stated. | 6-7 |
| Data collection process | 10 | The method of data extraction from reports and processes for obtaining and confirming data from investigators were described. | 7 |
| Data items | 11 | All variables for which data were sought, assumptions and simplifications were listed and defined . | 7 |
| Risk of bias in individual studies | 12 | Methods used for assessing risk of bias of individual studies were described. | 7 |
| Summary measures | 13 | The principal summary measures were stated. | 7 |
| Synthesis of results | 14 | The methods of handling data and combining results of studies were described, including measures of consistency. | 7 |

Page 1 of 2

| **Section/topic** | **#** | **Checklist item** | **Reported on page #** |
| --- | --- | --- | --- |
| Risk of bias across studies | 15 | Assessment of risk of bias that may affect the cumulative evidence was specified. | 7-8 |
| Additional analyses | 16 | Sensitivity and subgroup analyses were pre-specified and described. | 7-8 |
| **RESULTS** | | |  |
| Study selection | 17 | The numbers of studies screened, assessed for eligibility, and included in the review, with reasons for exclusions at each stage, ideally with a flow diagram were all given. | 9 and fig 1 |
| Study characteristics | 18 | For each study, present characteristics for which data were extracted and provide the citations. | 9 and table 1 |
| Risk of bias within studies | 19 | Data on risk of bias of each study was presented. | 12-13 |
| Results of individual studies | 20 | For all outcomes considered, present, for each study: (a) simple summary data for each intervention group (b) effect estimates and confidence intervals with a forest plot. | 13 |
| Synthesis of results | 21 | Results of meta-analysis was presented including confidence intervals and measures of consistency. | 13 |
| Risk of bias across studies | 22 | Results of assessment of risk of bias across studies was presented. | 15 |
| Additional analysis | 23 | Sensitivity and subgroup analyses were given. | 14-16 |
| **DISCUSSION** | | |  |
| Summary of evidence | 24 | The main findings including the strength of evidence for each main outcome were summarized; Their relevance to key groups was considered. | 17-18 |
| Limitations | 25 | Limitations at study and outcome level , and at review-level were discussed. | 19-20 |
| Conclusions | 26 | A general interpretation of the results in the context of other evidence, and implications for future research were Provided. | 21 |
| **FUNDING** | | |  |
| Funding | 27 | Sources of funding for the systematic review were described. | N/A |

*From:*  Moher D, Liberati A, Tetzlaff J, Altman DG, The PRISMA Group (2009). Preferred Reporting Items for Systematic Reviews and Meta-Analyses: The PRISMA Statement. PLoS Med 6(7): e1000097. doi:10.1371/journal.pmed1000097

For more information, visit: **www.prisma-statement.org**.

Page 2 of 2
